# Controlled Ovarian Hyperstimulation Protocol in Infertile Patients During the COVID-19 Pandemic

**DOI:** 10.3389/fphys.2021.732709

**Published:** 2021-09-27

**Authors:** Fei Li, HuiXia Zhang, WeiYi Shi, YiFang Wu, Ye Tian, YiHong Guo, HaiXia Jin, Gang Li

**Affiliations:** ^1^Center for Reproductive Medicine, The First Affiliated Hospital of Zhengzhou University, Zhengzhou, China; ^2^Center for Reproductive Medicine, The First People’s Hospital of Shangqiu, Shangqiu, China

**Keywords:** COVID-19 pandemic, controlled ovarian hyperstimulation, pituitary down-regulation, infertility, IVF/ICSI

## Abstract

**Objectives:** To explore the appropriate controlled ovarian hyperstimulation (COH) protocols in infertility patients who received the *in vitro* fertilization (IVF)/intracytoplasmic sperm injection (ICSI) treatments during the COVID-19 pandemic.

**Materials and Methods:** This retrospective cohort study evaluated the efficiency of the early follicular-phase long-acting GnRH-agonist long (EFLL) protocol (a new protocol developed by Chinese clinicians), prolonged pituitary down-regulation of EFLL protocol (Pro-EFLL), and the GnRH-ant protocol for couples meeting the study criteria between February 2020 and June 2020 who were treated by the First Affiliated Hospital of Zhengzhou University during the COVID-19 pandemic, and compared the pregnancy rates and miscarriage rates per fresh transfer cycle, number of retrieved oocytes, endometrial thickness on the day of hCG injection and the number of fertilized oocytes, mature oocytes, fertilized oocytes, and transferable embryos among the three protocols.

**Results:** We found that the prolonged pituitary down-regulation during the COVID-19 pandemic by utilizing a full-dose of GnRH-a administrated in infertility patients were no differences in clinical outcomes than other protocols, The prolonged pituitary down-regulation protocol and EFLL protocol were associated with a higher Endometrial thickness on the day of hCG injection (12.67 ± 2.21 vs. 12.09 ± 2.35 vs. 10.79 ± 2.38, *P* < 0.001), retrieved oocytes (14.49 ± 6.30 vs. 15.02 ± 7.93 vs. 10.06 ± 7.63, *P* < 0.001), mature oocytes (11.60 ± 5.71 vs. 11.96 ± 6.00 vs. 7.63 ± 6.50, *P* < 0.001), fertilized oocytes (9.14 ± 5.43 vs. 8.44 ± 5.34 vs. 5.42 ± 5.20, *P* < 0.001), and transferable embryos (4.87 ± 2.96 vs. 6.47 ± 5.12 vs. 3.00 ± 3.28 vs. *P* < 0.001) in the GnRH-antagonist protocol.

**Conclusion:** We recommend that patients start Gn injections 33–42 days after a pituitary downregulated full dose (3.75 mg) of gonadotropin-releasing hormone agonist during the COVID-19 pandemic, even a delay of 2–4 weeks does not affect the implantation rate. The study can provide a more detailed estimate and clinical management strategies for infertile couples during the COVID-19 pandemic.

## Introduction

The coronavirus disease-19 (COVID-19) pandemic started in late December 2019 in Wuhan, Hubei Province, China, and has since spread rapidly around the globe, with many countries being severely affected (Lai et al., 2020; Vermeulen et al., 2020). The disease as an acute respiratory infectious disease has been managed according to A class infectious diseases as stipulated in the Law of China. The Chinese government began enforcing social distancing, including restrictions on gatherings, public transportation and school closures limitations, including reproductive medicine procedures (Xia et al., 2020). During the special period of the epidemic, the European Society of Human Reproduction and Embryology (ESHRE) and the American Society for Reproductive Medicine (ASRM) have come together to jointly affirm the importance of continued reproductive research during the COVID-19 pandemic (Gianaroli et al., 2020; Veiga et al., 2020), committed to continuous monitoring of the effects of COVID-19 on reproduction, collecting data on infertility patients during the pandemic, and helping the majority of patients who seek treatment to ultimately become parents. However, there is no uniform standard on how to deal with infertile people and how to arrange medical treatment during this difficult time (Lupia et al., 2020; Pasquale et al., 2020; Veiga et al., 2020).

In the special period of the COVID-19 pandemic, it is necessary to develop or refine robust controlled ovarian hyperstimulation (COH) protocols to minimize exposure risks, to reduce the rate of cycle cancelations and to alleviate the financial and emotional burden of interrupting treatment for infertile couples due to the epidemic. To meet current needs, one full-dose depot of long-acting gonadotropin-releasing hormone agonist (GnRH-a) per COH cycle would be more suitable and convenient for women than short-acting GnRH-a injections or the GnRH antagonist protocol during the COVID-19 pandemic (Ben-Kimhy et al., 2020; Li F. et al., 2020), because there are fewer incidences of potential exposure. The early follicular-phase long-acting GnRH-agonist long (EFLL) protocol (a new protocol developed by Chinese clinicians) applies a pituitary downregulated full dose (3.75 mg) of dipherelin on days 2–4 of menstruation, and Gn starts 30–42 days later along with confirmation of the pituitary downregulation (Ying et al., 2019; Li F. et al., 2020). A series of studies has suggested its advantages in improving endometrial receptivity, embryo implantation and clinical pregnancy rates (Ren et al., 2014; Schisterman et al., 2020). It is worth emphasizing that the EFLL protocol was initially applied in a Chinese *in vitro* fertilization (IVF) center in 2016, and it has become the mainstream protocol in most reproductive medicine centers now in China (Li F. et al., 2020).

However, due to the interruption of medical treatment by COVID-19, many patients are affected by unexpected clinic closures (Sadeghi, 2020), and Gonadotrophins (Gns) would start > 42 days later along with confirmation of prolonged pituitary downregulation (pro-EFLL). Do these changes affect the outcome of assisted pregnancies in these infertile couples? Which controlled ovarian stimulation management strategies are most appropriate during the COVID-19 epidemic? In view of this, the aim of our study was to evaluate the appropriate COH protocol for infertility patients who received IVF/ICSI treatments during the COVID-19 pandemic. We gathered data comparing the clinical efficacy of the EFLL protocol, prolonged pituitary downregulation of the EFLL protocol and GnRH antagonist protocol before COH through collecting pregnancy rates and miscarriage rates per fresh transfer cycle, which are vital indicators for infertile couples (Vaegter et al., 2017; Veiga et al., 2020). To our knowledge, this is the first study to evaluate prolonged pituitary downregulation in infertile patients during the COVID-19 pandemic to provide a more detailed estimate and clinical management strategies for infertile couples.

## Materials and Methods

**FIGURE 1:**
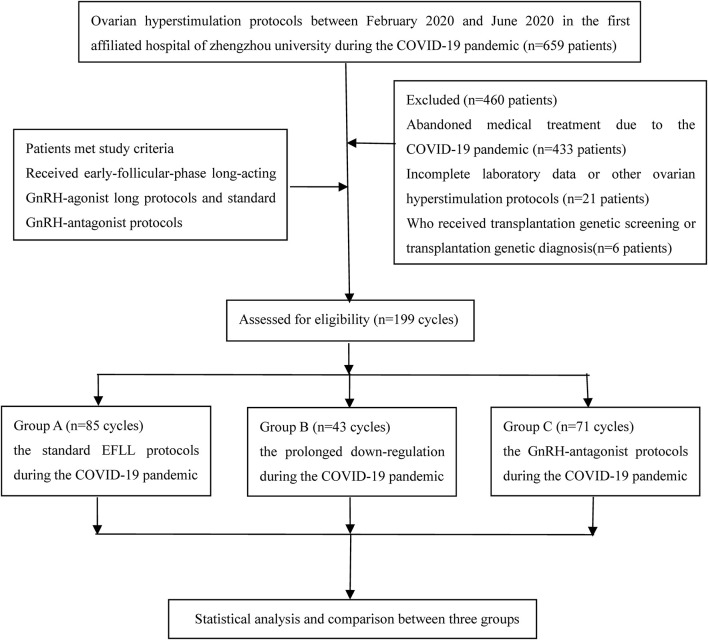
The flow chart.

This retrospective cohort study evaluated the efficiency of the EFLL protocol, the pro-EFLL protocol, and the GnRH-ant protocol for couples meeting the study criteria between February 2020 and June 2020 who were treated by the First Affiliated Hospital of Zhengzhou University during the COVID-19 pandemic. We screened eligible subjects and removed 433 patients who had abandoned treatment because of the COVID-19 pandemic, other ovarian hyperstimulation protocols and women who received transplantation genetic screening or transplantation genetic diagnosis or did not have complete laboratory data (e.g., Baseline data, Endocrine data, and Embryo data). Finally, the study analyzed clinical data from 199 cycles with IVF/ICSI in our reproductive medical center. The experimental materials in this study did not include identifiable part icipants data for the purpose of safeguarding patient privacy. This study was approved by the Ethics Committee of Reproductive Medicine Center, the First Affiliated Hospital of Zhengzhou University, China. Informed consent was waived, with approval from the ethics committee. A flow chart and the data processing procedure are listed in Figure 1.

### Early Follicular Phase Long-Acting GnRH Agonist Long Protocol

**FIGURE 2:**
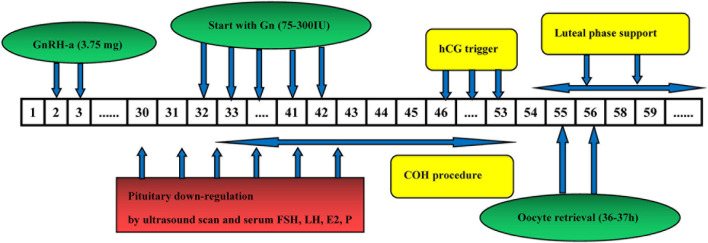
The flow chart of Early follicular phase long-acting GnRH agonist long protocol.

For patients undergoing the standard EFLL protocol, we administered 3.75 mg long-acting GnRH agonist (Diphereline, Ipsen, France) on days 2–3 of menstruation. Patients were monitored by sex hormones level and ultrasound measurements. The following criteria were used for down-regulation standard: No functional cysts and follicle sizes larger than 3–5 mm by ultrasound; LH < 5 IU/L, FSH < 5 IU/L, and *P* < 1 ng/mL. The initial dose of gonadotropin was administered on the basis of the woman‘s age, AFC, BMI, and ovarian response to stimulation. The trigger was administered with 2000 IU u-HCG (Livzon Pharmaceuticals) in combination with 250 μg r-human chorionic gonadotropin (hCG) (Merck Serono) when most dominant follicles are mature, the Oocytes were then retrieved 36–37 h after the trigger (Figure 2).

### Prolonged Pituitary Down-Regulation of EFLL Protocol

**FIGURE 3:**
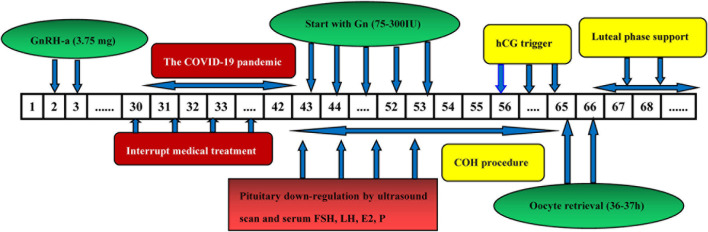
The flow chart of prolonged pituitary down-regulation of EFLL protocol.

The pro-EFLL protocol is analogous to the standard EFLL protocol as well, on days 2–3 of menstruation, we also administered 3.75 mg long-acting GnRH agonist (Diphereline, Ipsen, France). And all infertile patients were monitored by sex hormones level and ultrasound measurements, however, Gns would start >42 days later along with confirmation of prolonged pituitary down-regulation due to the COVID-19 interrupt medical treatment. The following process is the same as standard EFLL protocol (Figure 3).

### Gonadotropin-Releasing Hormone GnRH Antagonist Protocol

**FIGURE 4:**
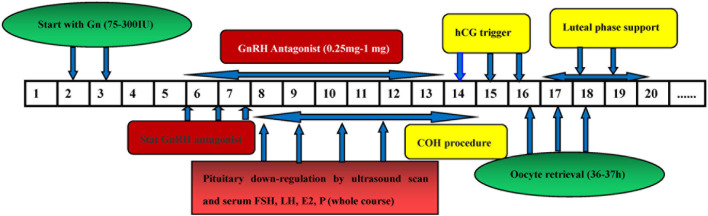
The flow chart of GnRH antagonist protocol. Figures 5, 6 comparison of treatment results among the three groups.

For the GnRH-ant protocol, COH was started with 72.5–300 IU gonadotropin (Puregon, Organon, Netherlands) on day 2–3 of the menstrual cycle. The initial dose of gonadotropin was administered on the basis of the woman’s age, AFC, BMI, and ovarian response to stimulation. A daily dose of 0.25–0.75 mg GnRH antagonist (Cetrotide, Pierre Fabre, Aquitaine Pharm International) was initiated on the sixth day of rFSH stimulation or when the lead follicle reached a mean diameter of 12–14 mm, and the gonadotropin was continued until the day of the trigger administration (5000 IU u-HCG, Livzon Pharmaceuticals or 250 μg r-hCG, Merck Serono in combination with 2000 IU u-HCG). The Oocytes were then retrieved 35–37 h after the trigger (Li F. et al., 2020; Figure 4).

### Follow-Up Procedure

We performed the follow-up through outpatient visits. The follow-up time began from their first clinical encounter and continued until the pregnancy outcome and miscarriage outcome occurred or the last date of this study, whichever occurred first.

### Statistical Analysis

All Data was analyzed using the software R (version 3.6.1) and the Statistical Package for Social Sciences (Version 22.0). The primary outcomes in this retrospective comparative study were pregnancy rates and miscarriage rates per fresh transfer cycle. The secondary outcomes included endometrial thickness, retrieved oocytes, mature oocytes, fertilized oocytes, and transferable embryos. Continuous variables were compared using one-way analysis of variance (ANOVA). Categorical variables were compared using the chi-square test or Fisher’s exact test. *P* < 0.05 was considered statistical significance.

## Results

Patients who met the study criteria between February 2020 and June 2020 and were treated at the First Affiliated Hospital of Zhengzhou University during the COVID-19 pandemic included 85 patients given the EFLL protocol, 43 patients given the pro-EFLL protocol, and 71 patients given the GnRH antagonist protocol. In the pro-EFLL protocol, the continuous pituitary downregulation time was between 43 and 63 days, 27 patients had a continuous pituitary downregulation time of 43–56 days, and the remaining patients it was between 57 and 63 days. There were no significant differences in the basic characteristics (Age, BMI, FSH, LH, E2, P PRL, AMH, AFC, TSH, FT3, FT4, Blood glucose) among the three groups. The EFLL protocol was associated with a shorter duration of pituitary downregulation (35.73 ± 2.87 vs. 56.56 ± 11.00, *P* < 0.001) and lower FSH levels on the Gn commencing day (3.40 ± 1.87 vs. 4.72 ± 1.90, *P* < 0.001) than the Pro-EFLL protocol. However, there were no significant differences in the pregnancy or miscarriage rates between the two groups (Table 1).

**TABLE 1:** Comparison of baseline parameters between the three groups.

Protocols	EFLL group (*n* = 85)	Pro-EFLL group (*n* = 43)	GnRH-ant group (*n* = 71)	*P*-value
Duration of pituitary down-regulation (days)	35.73 ± 2.87	56.56 ± 11.00^*a*^	/	<0.001
Age (years)	30.69 ± 4.38	31.26 ± 4.67	32.41 ± 5.97	0.108
BMI (kg/m2)	22.30 ± 2.97	23.08 ± 2.77	23.08 ± 3.57	0.229
Basal FSH (IU/L)	6.46 ± 1.60	6.13 ± 1.44	6.52 ± 1.65	0.427
Basal LH (IU/L)	5.38 ± 3.17	6.66 ± 5.16	5.17 ± 2.37^*b*^	0.049
Basal E2 (ng/L)	53.73 ± 121.79	56.39 ± 84.14	47.99 ± 43.07	0.878
Basal P (μg/L)	0.37 ± 0.49	0.52 ± 0.78	0.51 ± 0.32	0.147
PRL (ng/mL)	26.99 ± 42.82	18.37 ± 8.64	22.14 ± 38.07	0.421
AMH (ng/mL)	3.80 ± 3.05	3.84 ± 2.77	4.62 ± 4.02	0.269
AFC (numbers)	15.19 ± 6.55	15.35 ± 6.17	14.97 ± 7.58	0.958
TSH (mlU/mL)	2.19 ± 1.11	2.60 ± 1.31	2.35 ± 1.08	0.166
FT3 (pmol/L)	5.13 ± 0.85	5.30 ± 0.57	5.23 ± 0.56	0.398
FT4 (pmol/L)	11.45 ± 1.91	11.49 ± 1.49	11.75 ± 2.13	0.589
Blood glucose (mmol/L)	4.99 ± 0.42	5.03 ± 0.58	4.98 ± 0.48	0.864

*Data are shown as means ± standard deviation.*

*BMI, body mass index; FSH, follicular-stimulating hormone; LH, luteinizing hormone; E2, estradiol; P, progesterone; AMH, anti-Müllerian hormone; AFC, Antral Follicle Countin; TSH, thyroid stimulating hormone.*

*^*a*^P < 0.05, vs. early follicular phase long-acting GnRH agonist long protocol (Group A). ^*b*^P < 0.05, vs. prolonged GnRH-a down-regulation in fertility patients during the COVID-19 pandemic (Group B).*

Comparison of stimulation variables among the three groups revealed that the EFLL protocol was associated with a lower FSH level (3.40 ± 1.87 vs. 4.72 ± 1.90, *P* < 0.001) than the Pro-EFLL protocol on the Gn commencing day. We found that the EFLL protocol and the Pro-EFLL protocol were associated with a greater endometrial thickness (12.09 ± 2.35 vs. 12.67 ± 2.21 vs. 10.79 ± 2.38, *P* < 0.001), longer duration of Gn use (13.79 ± 1.96 vs. 13.02 ± 2.68 vs. 10.58 ± 2.51, *P* < 0.001), and a lower LH value (0.82 ± 0.81 vs. 1.08 ± 0.93 vs. 5.83 ± 0.99, *P* < 0.001) than the GnRH-ant protocol on the day of the hCG injection (Table 2).

**TABLE 2:** Comparison of stimulation variables between the three groups.

Protocols	EFLL group (*n* = 85)	Pro-EFLL group (*n* = 43)	GnRH-ant group (*n* = 71)	*P*-value
**On the Gn commencing day**				
FSH level (IU/L)	3.40 ± 1.87	4.72 ± 1.90^*a*^		<0.001
LH level (IU/L)	0.63 ± 0.40	0.64 ± 1.20		0.974
E2 level (IU/L)	14.74 ± 45.41	7.82 ± 6.02		0.323
P level (IU/L)	0.22 ± 0.12	0.19 ± 0.19		0.453
**On the day of hCG**				
Endometrial thickness (cm)	12.09 ± 2.35	12.67 ± 2.21	10.79 ± 2.38^*ab*^	<0.001
LH value (IU/L)	0.82 ± 0.81	1.08 ± 0.93	5.83 ± 0.99^*ab*^	<0.001
E2 value (IU/L)	3836.43 ± 2226.29	3276.20 ± 1980.63	3102.54 ± 2477.48	0.115
*P*-value (IU/L)	0.93 ± 0.51	0.97 ± 0.69	0.76 ± 0.57	0.102
Total dosage of Gn used (IU)	2569.12 ± 957.23	2485.47 ± 943.98	2536.44 ± 853.19	0.888
Duration of Gn used (days)	13.79 ± 1.96	13.02 ± 2.68	10.58 ± 2.51^*ab*^	<0.001

*Data are shown as means ± standard deviation or frequencies.*

*^*a*^P < 0.05, vs. early follicular phase long-acting GnRH agonist long protocol (Group A); ^*b*^P < 0.05, vs. prolonged GnRH-a down-regulation in fertility patients during the COVID-19 pandemic (Group B).*

**FIGURE 5:**
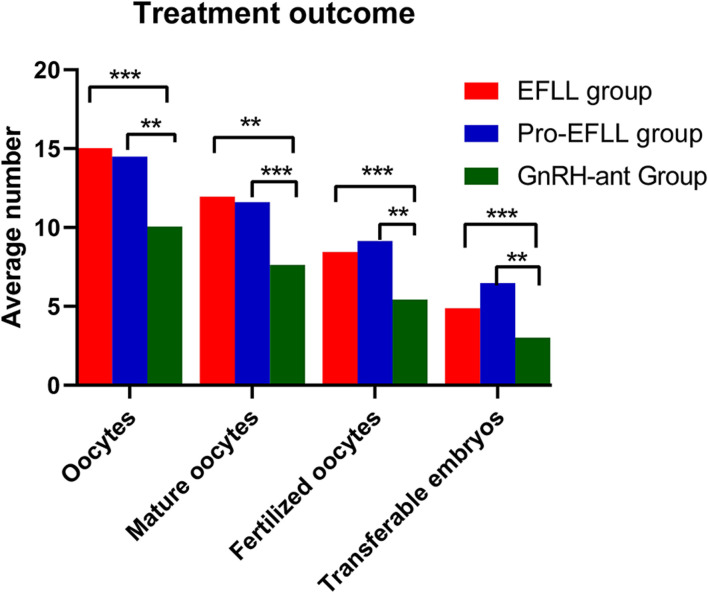
Comparison of oocytes retrieved, mature oocytes, fertilized oocytes, and transferable embryos among EFLL, Pro-EFLL, and GnRH-ant protocol. Values are presented as mean ± standard deviation. **Represents significant difference at *P* < 0.01, ***represents significant difference at *P* < 0.001.

**FIGURE 6:**
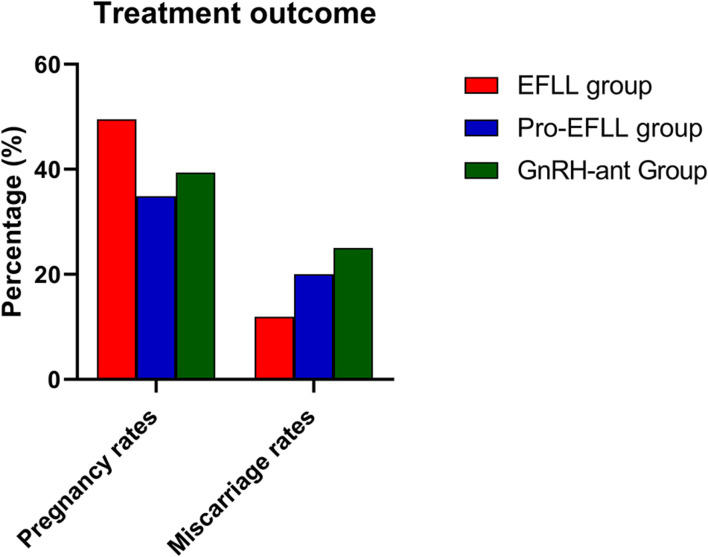
Comparison of pregnancy rates and miscarriage rates among EFLL, Pro-EFLL, and GnRH-ant protocol. Values are presented as frequencies.

We found that the GnRH-ant protocol was associated with a lower number of retrieved oocytes (10.06 ± 7.63 vs. 15.02 ± 7.93 vs. 14.49 ± 6.30, *P* < 0.001), mature oocytes (7.63 ± 6.50 vs. 11.96 ± 6.00 vs. 11.60 ± 5.71, *P* < 0.001), fertilized oocytes (5.42 ± 5.20 vs. 8.44 ± 5.34 vs. 9.14 ± 5.43, *P* < 0.001), and transferable embryos (3.00 ± 3.28 vs. 4.87 ± 2.96 vs. 6.47 ± 5.12, *P* < 0.001) than the EFLL protocol and the Pro-EFLL protocol; however, no statistically significant differences were seen for pregnancy rates (49.4 (42/85) vs. 34.9 (15/43) vs. 39.4 (28/71), *P* < 0.001) or miscarriage rates (11.9 (5/42) vs. 20.0 (3/15/34.9 (15/43) vs. 28.4 (28/71) per transfer cycle (Table 3). A comparison of treatment results among the three groups is shown in Figures 5, 6.

**TABLE 3:** Comparison of clinical outcomes between the three groups.

Protocols	EFLL group (*n* = 85)	Pro-EFLL group (*n* = 43)	GnRH-ant group (*n* = 71)	*P*-value
No. of oocytes	15.02 ± 7.93	14.49 ± 6.30	10.06 ± 7.63^*ab*^	<0.001
No. of mature oocytes	11.96 ± 6.00	11.60 ± 5.71	7.63 ± 6.50^*ab*^	<0.001
Oocyte maturation rates	0.82 ± 0.17	0.81 ± 0.18	0.76 ± 0.23	0.142
No. of fertilized oocytes	8.44 ± 5.34	9.14 ± 5.43	5.42 ± 5.20^*ab*^	<0.001
Fertilization rates	0.56 ± 0.23	0.63 ± 0.22	0.53 ± 0.27	0.120
No. Of transferable embryos	4.87 ± 2.96	6.47 ± 5.12^*a*^	3.00 ± 3.28^*ab*^	<0.001
Pregnancy rates per transfer (%)	49.4 (42/85)	34.9 (15/43)	39.4 (28/71)	0.229
Miscarriage rates (%)	11.9 (5/42)	20.0 (3/15)	25.0 (7/28)	0.358

*Data are shown as frequencies (percentages).*

*^*a*^P < 0.05, vs. early follicular phase long-acting GnRH agonist long protocol (Group A); ^*b*^P < 0.05, vs. prolonged GnRH-a down-regulation in fertility patients during the COVID-19 pandemic (Group B).*

## Discussion

Our findings indicate that prolonged pituitary downregulation during the COVID-19 pandemic by utilizing a full dose of GnRH-a administered to infertile patients was not associated with differences in pregnancy outcomes, such as pregnancy rates and miscarriage rates per fresh transfer cycle, among the three protocols. In addition, we also found that prolonged downregulation protocols and EFLL protocols can acquire more mature oocytes and transplantable embryos than GnRH-ant protocols. Furthermore, we found that these two protocols were associated with a greater endometrial thickness, longer duration of Gn use, and lower LH value than GnRH-ant protocols on the day of hCG injection. These strategies warrant further investigation. Considering that the ASRM and the ESHRE have no uniform standards on how to treat infertile people and how to arrange medical treatment during these difficult times (Esposito et al., 2020; Simopoulou et al., 2020; Veiga et al., 2020), to meet the current needs, our research results can provide a more detailed view of clinical management strategies for infertile couples during the COVID-19 pandemic.

In the special period of the epidemic, it is necessary to develop or refine robust COH protocols to minimize possible exposure. Our study showed that one full-dose depot of long-acting GnRH-a per COH cycle would be more suitable and convenient for infertile couples than GnRH antagonist and short-acting GnRH-a injections during the COVID-19 pandemic because there are fewer possible incidences of potential exposure (Ren et al., 2014; Li F. et al., 2020). Some studies previously observed that a pituitary downregulated full-dose may require a higher dose of gonadotropins for ovarian stimulation (Pan et al., 2019; Xu et al., 2020); however, our study showed that the total dosage of Gns used in our study was not statistically different among the treatment groups, which means a full dose of gonadotropins or prolonged pituitary downregulation would not drastically increase the economic burden for Infertile couples.

We recommend that patients start Gn injections 33–42 days after a pituitary downregulated full dose (3.75 mg) of gonadotropin-releasing hormone agonist during the COVID-19 pandemic. Even a delay of 2–4 weeks does not affect the implantation rate. This would significantly reduce the rate of cycle cancelations and greatly alleviate the financial and emotional burden of interrupting treatment for infertile couples due to the epidemic. Our study can provide a more detailed view of the clinical management strategies for infertile couples during the COVID-19 pandemic; however, these strategies warrant large-scale, prospective and multicenter clinical trials for confirmation in the future.

Our study showed that although the Pro-EFLL protocol was associated with a greater endometrial thickness than GnRH-ant protocols on the day of the hCG injection, the pregnancy rates were not significantly different among the groups. Similar findings were also reported in the study of Wang et al. (2017), Huang et al. (2018), and Liu Y. et al. (2019), and their conclusions are consistent with our findings. However, some studies in the past have shown that endometrial thickness is closely related to pregnancy rates (Haas et al., 2019; Liu W. et al., 2019). Gallos et al. (2018) analyzed 25,767 IVF cycles from the CARE Fertility Group in the United Kingdom and found that when the endometrial thickness was less than 5 mm, the live birth rate was 15.6%, and when the endometrial thickness of 10 mm, the live birth rate was gradually increased to 33.1%. It seems that the thicker the endometrium, the higher the pregnancy rates. We analyzed the reasons of these studies are inconsistent with our results may be caused by the following factors. First, endometrial thickness is not the only factor that affects the pregnancy rate since it is influenced by many factors, such as age, endometrial receptivity, and embryo quality (Zhao et al., 2014; Bu and Sun, 2015). Second, these studies did not take into account the effect of basal FSH, AMH, AFC, TSH, FT3, blood glucose or the E2 value on the day of the hCG injection per fresh transfer cycle when adjusting for covariates compared with our work, and previous studies have reported that these variables are related to pregnancy rates and miscarriage rates per fresh transfer cycle (Vaegter et al., 2017; Song et al., 2020). Third, the research populations are different, and there are physiological differences between ethnic Chinese and ethnic Europeans, the physiological difference between the two ethnic groups are reflected in many aspects, such as body mass index difference, altered ovarian morphology and functional changes, Genes associated with reproduction and fertility changes, which might cause different pregnancy outcomes (Mura et al., 1991; Lachance and Tishkoff, 2013; Dumesic et al., 2015).

Furthermore, the EFLL protocol can acquire more mature oocytes and transplantable embryos than the GnRH-ant protocol; however, no statistically significant effects were seen for pregnancy and miscarriage rates per fresh transfer cycle. Some reports suggest that it seemed more strongly impaired to endometrial receptivity by GnRH-a than GnRH-ant treatments, a study revealed that the gene expression profiles of endometrial cells following GnRH-ant treatment are more similar to those during natural cycles using microarray data (Chen et al., 2019), this finding may explain the phenomenon overall. However, some reports on endometrial receptivity have been inconsistent for the GnRH-ant and GnRH-a protocols, and studies have suggested that a full-dose dipherelin injection can be used to achieve long-term suppression of the GnRH agonist, and it can increase endometrial receptivity in patients, although the exact mechanism remains unclear (Lambalk et al., 2017; Wu et al., 2019), so further analysis is required in the future.

It is worth emphasizing that if patients require more COH cycles to achieve better cumulative live birth rates, our research shows that GnRH-a protocols is significantly superior to the GnRH-ant protocol. An animal model study suggested that follicles and embryos quality was significantly enhanced by increasing concentrations of GnRH-a and its receptor (Liu M. et al., 2018). One study showed that the optimal serum LH concentration on the commencing day of ovarian stimulation after downregulation with GnRH-a was 0.1 IU/L∼1 IU/L, and when serum LH levels are less than 0.1 IU/L, exogenous LH is required to increase the number of follicles and embryos. When the serum LH is greater than 1 IU/L, it has been proven to have adverse effect on embryo quality. Our study showed that serum LH levels on the commencing day of ovarian stimulation after downregulation were 0.63 ± 0.40 IU/L (EFLL Group) and 0.64 ± 1.20 IU/L (prolonged Group), which are similar to those in the study of Li G. et al. (2020). In view of these findings, we think that it is better to give patients a pituitary downregulated full dose (3.75 mg) of dipherelin on days 2–4 of menstruation during the COVID-19 pandemic.

The clinical value of this study is that it is the first to observe the effect of prolonged GnRH agonist downregulation on pregnancy and miscarriage rates per fresh transfer cycle in Chinese patients with infertility during the COVID-19 pandemic. The findings of this study should be helpful for developing clinical management strategies for infertile couples. However, there are some limitations in the present study (Jansen et al., 2020; Niehus et al., 2020): (1) Retrospective cohort studies are always associated with selection bias issues, with selection bias being introduced, one might expect the result to be skewed in some ways. (2) In this study, our research subjects were all Chinese patients with infertility being given IVF/ICSI treatments during the COVID-19 pandemic. Therefore, there is a certain deficiency in the universality and generalizability of the research. (3) Because we excluded women who received transplantation genetic screening or other ovarian hyperstimulation protocols, the findings of this study cannot be used for these groups of people.

In conclusion, in the special period of the COVID-19 pandemic, prolonged pituitary downregulation by utilizing a full dose of GnRH-a administered to infertility patients showed no differences in clinical outcomes, such as pregnancy or miscarriage rates per fresh transfer cycle, among the different protocols. The prolonged downregulation protocol and the EFLL protocol can acquire more mature oocytes and transplantable embryos than the GnRH-ant protocol. We recommend that patients start Gn injections 33–42 days after a pituitary downregulated full dose (3.75 mg) of gonadotropin-releasing hormone agonist during the COVID-19 pandemic. Even a delay of 2–4 weeks does not affect the implantation rate. Our study can provide a more detailed estimate and clinical management strategies for infertile couples during the COVID-19 pandemic; however, these strategies warrant large-scale, prospective and multicenter clinical trials for confirmation in the future.

## Data Availability Statement

The original contributions presented in the study are included in the article/supplementary material, further inquiries can be directed to the corresponding author/s.

## Ethics Statement

This study was followed the Declaration of Helsinki Guideline and approved by the Ethics Committee of Reproductive Medicine Center, the First Affiliated Hospital of Zhengzhou University, China. Ethical code: ZZDX-2020-T260-16.

## Author Contributions

GL, HJ, and FL conceived of and designed the experiments. GL, YT, HZ, and WS selected and supervised suitable patients. GL, YW, and FL obtained basic clinical data including age, body mass index, FSH, LH, estradiol, progesterone, and AMH levels, total dosage of gonadotropin used, duration of gonadotropin use, oocyte number, and live birth rate per transfer. GL provided overall supervision. GL, HZ, and FL drafted the manuscript. All authors reviewed this manuscript.

## Conflict of Interest

The authors declare that the research was conducted in the absence of any commercial or financial relationships that could be construed as a potential conflict of interest.

## Publisher’s Note

All claims expressed in this article are solely those of the authors and do not necessarily represent those of their affiliated organizations, or those of the publisher, the editors and the reviewers. Any product that may be evaluated in this article, or claim that may be made by its manufacturer, is not guaranteed or endorsed by the publisher.
